# Development and validation of the Health Literacy Index for the Community for the Korean National Health and Nutrition and Examination Survey

**DOI:** 10.4178/epih.e2024061

**Published:** 2024-07-10

**Authors:** Junghee Yoon, Soo Jin Kang, Mangyeong Lee, Juhee Cho

**Affiliations:** 1 Department of Clinical Research Design and Evaluation, SAIHST, Sungkyunkwan University, Seoul, Korea; 2 Patient-Centered Outcomes Research Institute, Samsung Medical Center, Seoul, Korea; 3 Department of Nursing, Daegu University, Daegu, Korea; 4 Center for Clinical Epidemiology, Samsung Medical Center, Sungkyunkwan University School of Medicine, Seoul, Korea; 5 Cancer Education Center, Samsung Medical Center, Seoul, Korea

**Keywords:** Health literacy, Surveys and questionnaires, Korean National Health and Nutrition and Examination Survey

## Abstract

**OBJECTIVES:**

We developed and validated the Health Literacy Index for the Community (HLIC) to assess the health literacy of the Korean population within the framework of the Korean National Health and Nutrition and Examination Survey.

**METHODS:**

The HLIC was developed through (1) defining the conceptual framework and generating the item pool and (2) finalizing the items and identifying the cut-off value. Interviews were conducted to examine items’ face validity, and a cross-sectional survey was performed to analyze the item-response theory and Rasch models to investigate the instrument’s psychometric properties.

**RESULTS:**

In this study of 1,041 participants, most had no difficulty understanding health information; however, 67.9% struggled to assess the reliability of health information from the Internet or media. A 4-factor structure was identified through factor analysis, leading to the exclusion of some items. This resulted in 10 items across 4 domains: (1) disease prevention, (2) health promotion, (3) health care, and (4) technology and resources. The HLIC demonstrated good internal consistency, with a Cronbach’s α of 0.87. It also showed high test-retest reliability and correlations with other health literacy instruments. A socio-demographic analysis of the HLIC revealed disparities in health literacy across various age groups, education levels, and income brackets.

**CONCLUSIONS:**

The HLIC was developed to systematically measure health literacy in Korea’s general population. Its simplicity and conciseness ensure reliability and validity and improve its accessibility, making it particularly suitable for the broader Korean population, including those with lower literacy levels.

## GRAPHICAL ABSTRACT

[Fig f2-epih-46-e2024061]

## Key Message

This study developed and validated the Health Literacy Index for the Community (HLIC) to assess the health literacy of Koreans, finding that many had difficulties to assess its reliability from Internet or media sources, and disparities in health literacy were observed across age, education level, and income. The HLIC demonstrated strong reliability and validity, highlighting its utility for identifying health literacy gaps and informing targeted interventions in Korea.

## INTRODUCTION

Health literacy, which is a critical factor for informed health decision-making, refers to the ability to find, understand, evaluate, and apply health-related information in daily life [1]. This understanding is crucial for promoting healthier lifestyles [2,3]. Serving as a pivotal determinant of health outcomes and social health disparities, health literacy predicts an individual’s overall health status [2,3]. Inadequate health literacy is associated with poor health outcomes, such as lower vaccination rates and cancer screening, an increased incidence of chronic diseases, and more hospitalizations and use of emergency care [4,5]. As healthcare systems advance in complexity and public health challenges emerge, there has been a growing emphasis on health literacy. The World Health Organization recommends monitoring and improving health literacy as a core strategy for promoting community health and reducing health disparities [6]. Many countries have developed their own measures, implemented health literacy initiatives, and outlined policy directions [7-10].

In Korea, despite a high overall literacy rate due to widespread educational attainment [11], a significant segment of the population exhibits limited health literacy. The Korean government recently included health literacy as an overarching goal of the Healthy Plan 2030 for community health [12,13] and decided to assess the health literacy of the Korean population from 2023 using the Korean National Health and Nutrition and Examination Survey (KNHANES), a national surveillance system to produce nationwide statistics regarding health outcomes [14,15].

To effectively assess health literacy at the population level, it is essential to develop a valid and reliable instrument tailored to community measurements. Measurement instruments for community health must align with national health outcomes and organizational health literacy to ensure relevance and practical applicability in promoting individual and community well-being. The European Health Literacy Survey (HLS-EU-Q47) [16], a widely used tool, does not evaluate competencies for managing health issues through civic engagement, which are essential for understanding social determinants of health and promoting well-being [17,18].

Studies using the HLS-EU-Q47 in Korea have revealed cultural differences in health values and decisions [19] and the scales were often age-specific or limited in scope [20-22]. Korea is facing challenges in encouraging people to actively manage their health as informed health agents. However, the HLS-EU-Q47 addresses the accessibility of health information, whereas in Korea, there is a greater need to emphasize understanding and utilizing diverse information sources. Currently, no rigorously validated health literacy instrument exists for the general Korean population [23-25].

Thus, we developed and validated the Health Literacy Index for the Community (HLIC), a tool specifically tailored to assess the general health literacy of the Korean population, within the KNHANES framework.

## MATERIALS AND METHODS

**Figure 1.**
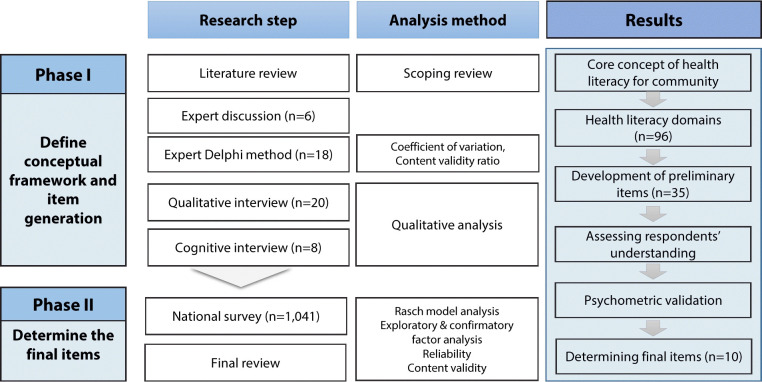
Development process of the Health Literacy Index for the Community.

The HLIC was developed from January 2022 to August 2022, as outlined in Figure 1. In the first phase, we developed a conceptual framework and generated an item pool and an initial version of the HLIC. In the second phrase, we finalized the items, validated their psychometric properties, and identified the cut-off value.

### Phase 1. Definition of the conceptual framework and item generation

The conceptual framework and item pool of the HLIC were established through a comprehensive literature review, 2 rounds of Delphi surveys, and qualitative interviews with the general population.

#### Literature review

Our literature review followed the PRISMA-ScR guidelines for scoping reviews [26]. Existing measures of health literacy, such as the HLS-EU-Q47 [16], Korean Health Literacy Instrument [27], Chew’s Measurement [28], Korean Health Literacy Scale [29,30], and eHealth Literacy Scale [31,32], were reviewed.

#### Expert discussion and expert Delphi method

We asked a panel of experts (n= 6) representing diverse health specialties to confirm the conceptual framework and the operational definitions of the HLIC, considering different concepts of health literacy in various healthcare environments.

Then, to develop an initial version of the HLIC, 2 rounds of a Delphi survey were conducted between April 2022 and June 2022. In the first round, the experts were asked to assess the face and content validity of 96 items. We invited a diverse panel of experts (n= 18) comprising 11 professors, 3 physicians, 3 nurses, and 1 researcher, each with over 3 years of experience in health literacy or health communication. The experts evaluated the appropriateness and relevance of the domains, the importance of the generated items, and the response scale and encouraged them to suggest any missed domains or items. A 4-point Likert scale was used, and the coefficient of variation and content validity ratio (CVR) were calculated. In the second round, the experts reevaluated the items for their importance and relevance to health literacy. In this round, 15 experts of 18 experts participated, and 13 completed the survey.

#### Qualitative interview

Qualitative interviews (n = 20) were conducted to gauge the general population’s perception and utilization of health information (including health literacy-related experiences), ensuring alignment with our conceptual framework. When developing a measurement tool, conducting interviews with the target population is crucial [33], with members of the population describing their experiences in lay language to identify essential construct components and build content validity, ensuring that the tool accurately reflects their experiences. The interviews were conducted face-to-face or over the phone with 20 adults stratified by age, gender, and chronic disease prevalence [34].

#### Cognitive interviews

Cognitive interviews (n= 8) were then conducted, either face-to-face or online, to assess the participants’ comprehension of the initial 35 items of the HLIC. We examined the participants’ memory retrieval, cognitive processes, understanding of terminology, response options, formats, and selection of responses. Each item was evaluated by at least 5 individuals.

### Phase 2. Determination of the final items and identification of the cut-off value

#### National survey

We conducted a national survey to reduce the number of items, finalize the selection, and identify the cut-off value. A representative sample of Korean adults aged 19 years and older was invited to the survey using a multistage stratified sampling method based on age, gender, area of residence, and socioeconomic status (SES) from July 20, 2022 to July 31, 2022. This sampling method was the same as that used for the KNHANES 2021. Considering the digital divide, the survey was administered both online and offline. The participants aged under 55 years completed the survey online using a computer-assisted self-interviewing system, while those aged 55 and above were surveyed face-to-face. Socio-demographic information such as age, gender, marital status, education, monthly family income, employment status, chronic disease prevalence, and health information experience were collected. Within 1-3 weeks after the first survey, about 10% of the participants (n= 101) with an average age of 47.0 years (standard deviation [SD], 13.0) were asked to complete the same items to assess test-retest reliability. Exploratory factor analysis (EFA) and confirmatory factor analysis were used to evaluate content, construct, convergent, and discriminant validity. Internal consistency and test-retest reliability were assessed to ensure the reliability of the HLIC. To examine criterion and convergent validity, the HLS-EU-16 and the Newest Vital Sign (NVS) for functional health literacy were included [35].

#### Final review

Finally, to ensure the robustness and applicability of the HLIC, we consulted the Advisory Committee of the Korea Centers for Disease Control and Prevention, Ministry of Health and Welfare, and the Korea Institute for Health Promotion with 7 experts who participated in the Delphi survey. The cut-off value for HLIC was determined using the area under the receiver operating characteristic (ROC) curve. Individuals were categorized as having adequate or inadequate health literacy.

### Statistical analysis

Descriptive statistics were used to report the participants’ characteristics and explore the item distribution. To assess construct validity, EFA using principal component analysis with varimax rotation was performed. We considered eigenvalues above 1 and factor loadings greater than or equal to 0.40, focusing on items that loaded the highest on a particular factor to confirm the potential underlying factors. For an efficient instrument with improved item information, item response theory (IRT), which provides a richer description of the performance of each item, was used. The Rasch model was applied to estimate item difficulty and to determine the items that best aligned with the latent variables and constructs of the HLIC. Additionally, we examined mean square (MNSQ) fit statistics, including infit and outfit statistics, to assess the adequacy of item fit within the model.

To examine reliability, internal consistency was calculated using the Cronbach’s α coefficient, with 0.6 as an acceptable cut-off value. For test-retest reliability, we used the intraclass correlation coefficient (ICC). Typically, ICC values less than 0.50 indicate poor reliability, values between 0.50 and 0.75 indicate moderate reliability, values between 0.75 and 0.90 indicate good reliability, and values greater than 0.90 indicate excellent reliability [36]. We performed a bivariate analysis between the HLIC and HLS-EU-16 scores to examine convergent and discriminant validity, using Pearson correlation coefficients.

Total scores were calculated by summing responses for the selected items. Optimal cut-off scores for the HLIC were determined using area under the curve (AUC) calculations and ROC analysis, with the HLS-EU-Q16 cut-off as the reference standard for ROC curves [37]. Each item of the HLS-EU-Q16 was converted into a dichotomous response, where “very difficult” and “difficult” were coded as 0, and “easy” and “very easy” were coded as 1. The final score for each participant was determined by summing the scores (0 or 1) across the 16 items. Scores ranging from 0 points to 8 points were categorized as reflecting an inadequate level of health literacy, scores from 9 points to 12 points as a problematic level, and scores from 13 points to 16 points as a sufficient level. Therefore, in this study, ROC curves were generated using a reference standard derived from the HLS-EU-Q16 with a cut-off value of 8.

The significance level was set at p-value < 0.05 (2-sided). All statistical analyses were performed using Stata version 14 (StataCorp., College Station, TX, USA), Mplus 8.0 (https://www.statmodel.com/), and jMetrik (https://itemanalysis.com/jmetrik-download/).

### Ethics statement

The study was approved by the Institutional Review Boards of Samsung Medical Center (IRB 2022-02-013-005).

## RESULTS

### Phase 1. Definition of the conceptual framework and item generation

#### Literature review

The scoping review process is illustrated in Supplementary Material 1. Based on a scoping review, the U.S. Centers for Disease Control and Prevention recently redefined health literacy, emphasizing the ability to use health information and the responsibility of organizations in supporting health literacy [38]. The new definitions focus on making “well-informed” decisions, highlight organizational responsibility, and connect health literacy to health equity. In this study, we adopted these revised definitions to align with contemporary understandings of health literacy, and we established the core conceptual framework of the HLIC, adopting Sorensen’s health literacy dimensions, encompassing 3 health domains and 4 cognitive domains (12 subscales), one of the most widely used concepts for health literacy [16], The selection of the 4 domains for the HLIC was based on both theoretical considerations and practical relevance to the Korean context.

#### Expert discussion and expert Delphi method

In the first round, according to the table of critical values from the revisited methods of calculating Lawshe’s CVR [39], a CVR of 0.44 or higher (n= 18) signified good content validity. In this step, 90 items met the agreement threshold. We set a CVR of 0.70 or higher for stricter selection, resulting in 65 items. In the second round, 27 items achieved high consensus, and 5 additional items were suggested by 3 or more experts as potentially useful.

Additionally, after an internal discussion, 3 items suggested by the research team were included in the initial version of the HLIC, which consisted of 35 items in 4 domains: disease prevention (7 items), health promotion (4 items), health care (19 items), and technology and resources (5 items). A 4-point Likert response scale (ranging from “very easy” to “very difficult”) was prepared. The research team further reviewed these items to evaluate their relevance, simplicity, and clarity. During this step, we determined that the short survey had to omit cognitive domains to reduce the burden on respondents and improve participation rates, focusing instead on essential health literacy metrics for efficient data collection.

#### Qualitative interviews

In the interviews, we found that people (mean ± SD = 49.8 ± 16.9 years; women= 45.5%; education level below high school= 40.0%; employed= 45.0%) tended to need health information when they have health problems. Most participants obtained health information through the internet, but participants with low literacy relied on human elements who had experienced similar situations, and some reported high confidence in health information because they did not know what was wrong with their health. Some of the above results should be referred to in the context of findings previously published in a related study [34].

#### Cognitive interviews

The 8 participants’ mean± SD age was 47.9± 16.4 years, 62.5% were women, and 62.5% had less than a high school education. The results showed that the participants generally comprehended the HLIC well, with the exception of 3 items (Supplementary Material 2). For validity, we entrusted the Federation of Korean Language and Cultural Center (https://kplain.kr/) with grammar, vocabulary, spacing, and so forth. After reviewing, the initial response options were changed from “very easy-easy-difficult-very difficult” to “always-sometimes-rarely-never” to ensure more suitable answer choices.

#### National survey

In our study of 1,041 participants, the mean age was 47.1± 16.3 years, 24.0% were 60 years or older, and 43.6% had less than a high school education (Table 1). As presented in Table 2, 70-90% of participants responded “always” or “sometimes,” indicating that they had no difficulty in understanding, judging, and utilizing health information, to 35 items. However, 67.9% of the respondents answered “always” or “sometimes” to the item assessing whether they could assess the reliability of health information obtained from the internet or media (e.g., television, YouTube).

EFA revealed a construct consisting of 4 factors with an eigenvalue greater than 1.00. Low-factor loadings (< 0.40) or loadings onto 2-factor constructs were eliminated. Thus, 7 items that loaded onto the 2-factor solutions were removed (Table 2). Although the item “Can you manage post-treatment symptoms relief and side effect prevention on your own?” had a loading of 0.49 on factor 1, it was excluded following deliberation after discussion (Table 2) [40].

Item fit was evaluated using the outfit and infit MNSQ statistics. Most items showed acceptable fit through IRT and Rasch model analyses, but 8 items were misfits (MNSQ > 1.3 or < 0.7). Item difficulty mostly ranged from -0.96 to 1.16, with 9 items having the same difficulty level. Based on the misfits, difficulty levels, and content validity reviewed by psychometricians and content experts, 9 items were excluded. Additionally, after expert discussions on redundancy and relevance, 8 more items were excluded (Table 2).

Finally, we decided on 10 items based on response frequency and differences in the results analyzed by subgroups, such as age and income, item difficulty, fit indices, and content validity for use in the KNHANES (Table 3). The final version of the HLIC demonstrated good internal consistency (overall Cronbach’s α= 0.87; 0.72-0.78 for subtotals).

Regarding test-retest reliability, the ICCs of each item ranged from 0.33 to 0.58; however, the overall correlation coefficient was good (r = 0.67). The HLIC was strongly associated with the eHEALS (r = 0.67), moderately associated with the DHTL-AQ (r= 0.45), and weakly associated with the NVS (r= 0.22). Regarding the convergent validity of the HLIC, a moderate correlation (0.37 to 0.54) was observed between each item in the HLIC and similar items in the HLS-EU-Q16 (Table 4). The AUC of the HLIC score was 0.88 (95% confidence interval [CI], 0.85 to 0.91). The cut-off value for the HLIC was 28 out of 40 (Supplementary Material 3).

Table 5 presents a comparison between higher and lower health literacy based on the cut-off value of the HLIC. Approximately 18.5% of individuals under 30 years of age exhibited low health literacy, in contrast to 35.2% of those in their 60s or older. Low health literacy was observed in 54.2% of participants with a middle school education or less, compared with only 16.5% of college graduates and 13.5% of those with graduate degrees. Regarding family income, 48.0% and 35.8% of those earning US$2,000 or less per month had low health literacy, in contrast to 14.3% and 15.7% among earners of US$4,000 to less than US$5,000 and US$5,000 or more, respectively.

#### Final review

Finally, after consultation with relevant experts, departments, and the research team, the final version of the HLIC consisted of 3 items in disease prevention, 1 item in health promotion, 4 items in health care, and 2 items in resource utilization (Supplementary Material 4)

## DISCUSSION

The HLIC was developed to systematically measure health literacy in Korea’s general population. Its simplicity and conciseness ensure reliability and validity and improve its accessibility, making it particularly suitable for the broader Korean population, including those with lower literacy levels.

Developing a straightforward tool that aligns with this definition and encompasses diverse aspects of health literacy is a complex endeavor [41]. In response to these challenges, we undertook a comprehensive literature review, Delphi surveys, and in-depth interviews, establishing a robust conceptual framework and diverse item pool. This was followed by a national survey for final item selection and psychometric validation. The HLIC’s development involved collaboration with government stakeholders, health professionals, literacy experts, and the general population, ensuring a broad consensus.

Despite its brevity, the HLIC’s 10 items comprehensively cover key health literacy domains, aligning with indicators from Healthy People 2030 (HP 2030) in the United States [12] and the central domains of the HLS-EU-Q47 in Europe [16]. HLIC covers the key dimensions of Sorensen’s health, including disease prevention, health promotion, and healthcare. It also included items on healthcare communication and decision-making based on online information, reflecting HP 2030’s focus on health communication and information technology. The instrument also mirrors key aspects of the HLS-EU-Q47, HLS-EU-Q16, and HLS-EU-Q12 [41,42], such as evaluating mental health risk judgment and health behavior effects. It shows high internal consistency and strong criterion and discriminant validity, as evidenced by its use of the HLS-EU-Q16 [37].

The HLIC’s suitability for the Korean context and consistency with international standards makes it a valuable tool in national health policy and global health literacy research. Its concise design, coupled with comprehensive coverage, enables efficient health literacy assessment and is ideal for integration into large-scale surveys and monitoring systems. Furthermore, the inclusion of the technology and resources domain is particularly relevant in today’s digitally driven healthcare environment. Future research could explore its applicability in other cultural contexts and its potential for adaptation to other languages and healthcare systems. In addition, longitudinal studies using the HLIC could provide valuable data on the evolution of health literacy, especially in the face of rapidly changing healthcare landscapes and technological advancements.

Health literacy assessments typically involve self-reported and knowledge-based measures. O Neill et al. [43] noted that self-administered measures for health literacy are more scalable, practical, and easily incorporated into surveys. However, knowledge-based items may cause psychological stress, especially for those with low literacy, and may result in measurement bias because these items are more likely to focus on intelligence-related competencies, such as reading and doing math, and are separate from health literacy [44]. Future research should clarify the extent to which knowledge-based health literacy is necessary, the areas to be measured, and how to measure it.

In our study, approximately 22.2% of the general population had low health literacy levels. This is a relatively low proportion compared with the results of a previous study [45]. A cross-sectional study with a nationally representative sample of Korean adults found that approximately 61% of participants had inadequate health literacy. However, this study assessed health literacy using the NVS in 2016, which is knowledge-based measure [45]. A possible explanation for this result may be that the education level of older adults has increased since 2016, owing to the expansion of compulsory education. Although health literacy has increased, we found that people 60 years or older and those with a low SES had lower health literacy levels than younger and high-SES people.

Low health literacy is related to difficulties in finding and understanding information, which has a significant impact not only on decision-making during treatment but also on disease prevention and health promotion [46]. These health-literacy-related problems can lead to a vicious cycle in conjunction with issues such as social roles, economic disparities, and conflicts within the family; therefore, interventions based on appropriate circumstances are necessary [47]. Health literacy is the missing link in explaining the relationship between health education and health outcomes for improving both the overall ability of individuals to effectively manage their health conditions and the community [48,49]. Therefore, health literacy should be evaluated, particularly in vulnerable populations, such as older, less educated, and lower-income segments. This also means that when designing a health literacy index for the community, the specific health concepts, terminology, and evolving health issues and preferences relevant to the target population should be prioritized and identified, while ensuring that the instrument is effective and accessible.

This study has several limitations. First, the survey for psychometric validation was conducted online and face-to-face, considering digital literacy and social distancing measures associated with the coronavirus disease 2019 (COVID-19) pandemic. This may have affected the validity and reliability of the instrument. However, we considered the training of the personnel who administered the interviews, standardization of question types, and other measures to ensure uniformity between the 2 modes. Second, the participants’ healthcare experiences may have varied due to the timing of the COVID-19 pandemic. As health literacy is influenced by the healthcare system, public health infrastructure, cultural context, and the concept of health changes, the HLIC needs to be updated on an ongoing basis.

Developed through rigorous research and stakeholder collaboration, the HLIC is an innovative tool that captures the multifaceted nature of health literacy. It is valuable for communities, including vulnerable groups, and versatile for diverse cultural contexts, making it relevant globally. This study provides insights into health literacy and lays the groundwork for future research and policies to enhance health literacy and equity, especially among vulnerable populations.

## Figures and Tables

**Figure f2-epih-46-e2024061:**
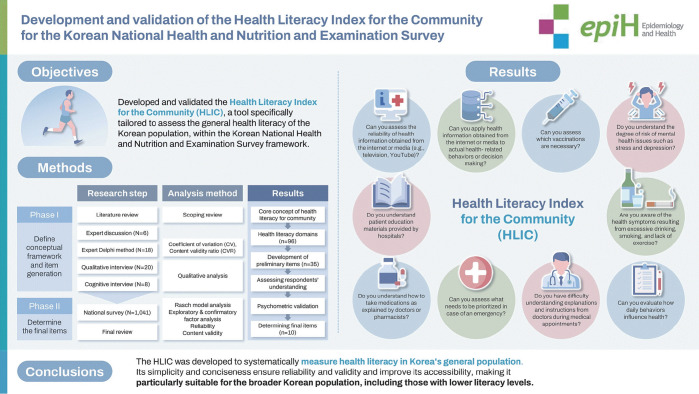


**Table 1.** Participants’ characteristics (n=1,041)

Characteristics	n (%)
Gender, men	524 (50.3)
Age, mean±SD [range] (yr)	47.1±16.3 [18-84]
19-29	184 (17.7)
30-39	185 (17.8)
40-49	212 (20.4)
50-59	210 (20.2)
≥60	250 (24.0)
Education	
≤ Middle school	131 (12.6)
High school	323 (31.0)
College/University	498 (47.8)
Graduate school or higher	89 (8.5)
Marital status	
Single	357 (34.3)
Married	609 (58.5)
Divorced/widowed	75 (7.2)
Area of residence	
Capital/metropolitan area	462 (44.4)
Local area	579 (55.6)
Working status (yes)	716 (68.8)
Monthly family income (US dollar)	
≤1,000	50 (4.8)
1,000-<2,000	109 (10.5)
2,000-<3,000	157 (15.1)
3,000-<4,000	136 (13.1)
4,000-<5,000	126 (12.1)
≥5,000	274 (26.3)
Unknown	189 (18.2)
Comorbidities (none)^1^	580 (55.7)
Hypertension	205 (19.7)
Diabetes	104 (10.0)
Hyperlipidemia	101 (9.7)
Rheumatic disease, arthritis, osteoporosis	67 (6.4)
Overall self-reported health status during the past 7 days	
Mean±SD^2^	4.6±1.2
Low (≤3)	192 (18.4)
Moderate (4-5)	617 (59.3)
High (≥6)	232 (22.3)
Overall quality of life during the past 7 days	
Mean±SD^2^	4.6±1.2
Low (≤3)	184 (17.7)
Moderate (4-5)	614 (61.6)
High (≥6)	216 (20.7)

SD, standard deviation.

1 Multiple responses.

2 Measured on a global scale (1=very poor, 7=excellent).

**Table 2.** Factor loading and decisions for the initial 35 items

Domains	Items	n (%)^1^	Factor loading values				Decision
			1	2	3	4	
Disease prevention							
Vaccination	Do you comprehend precautions before and after vaccination?	979 (94.4)	0.45	-	0.58	-	Deleted^3^
	Can you assess which vaccinations are necessary?^2^	876 (84.2)	-	-	0.57	-	
Health screening	Can you read and comprehend the results of health checkups for yourself and your family?	870 (85.6)	-	-	0.53	-	Deleted^4^
	Can you determine which items in the national health checkup are applicable to you?	862 (82.8)	-	-	0.59	-	Deleted^5^
Mental health	Do you understand the degree of risk of your mental health issues such as stress and depression?^2^	818 (78.6)	-	-	0.58	-	-
	Can you easily find information on activities beneficial for mental health (e.g., yoga, meditation, walking)?	853 (81.9)	-	0.41	0.45	-	Deleted^3^
Disease prevention	Are you aware of the health signs resulting from excessive drinking, smoking, and lack of exercise?^2^	864 (83.0)	-	-	0.59	-	-
Health promotion							
Environment	Do you comprehend information about the impact of the surrounding environment (fine dust, air quality, water quality, housing, etc.) on health?	876 (84.2)	-	-	0.60	-	Deleted^4^
	Can you appropriately respond to environmental risk alerts (e.g., fine dust, yellow dust, heatwaves, cold waves) in daily life?	830 (79.7)	-	-	0.63	-	Deleted^4^
Healthy behavior	Do you participate in health promotion programs?	878 (84.3)	-	0.52	0.47	-	Deleted^3^
	Can you evaluate how daily behaviors influence health?^2^	866 (83.2)	-	-	0.57	-	-
Healthcare							
Communication with health professionals	Do you have difficulty understanding explanations and instructions from doctors during medical appointments?^2^	986 (94.7)	0.63	-	-	-	-
	Can you engage in conversations with doctors during medical appointments without assistance?	955 (91.7)	0.68	-	-	-	Deleted^5^
	Do you understand information provided by medical staff or pharmacists when using hospitals or pharmacies?	959 (92.1)	0.67	-	-	-	Deleted^4^
	Can you follow the doctor's or pharmacist’s instructions for taking prescribed medications?	982 (94.3)	0.73	-	-	-	Deleted^5^
	Do you comprehend documents or instructions provided by medical institutions and health centers?	935 (89.8)	0.69	-	-	-	Deleted^4^
Emergency	Can you find a place or person to seek help from in case of an emergency?	885 (85.0)	0.44	-	-	0.50	Deleted^3^
	Can you determine what to do in case of an emergency?	803 (77.1)	-	-	-	0.78	Deleted^5^
	Can you assess what needs to be prioritized in case of an emergency?^2^	784 (75.3)	-	-	-	0.78	-
Pharmacy and medical information	Can you independently make appointments at comprehensive hospitals when you are unwell?	884 (84.9)	0.54	0.42	-	-	Deleted^3^
	Can you find places for assistance, such as pharmacies or hospitals, on holidays or public holidays when you have health issues?	841 (80.8)	0.43	0.42	-	0.43	Deleted^3^
	Can you handle reception, payments, and tests at hospitals without assistance?	944 (90.7)	0.68	-	-	-	Deleted^5^
	Can you describe your current health status (e.g., medical history, medications, symptoms)?	934 (89.7)	0.60	-	-	-	Deleted^5^
Medication	Do you understand how to take medications as explained by doctors or pharmacists?^2^	979 (94.0)	0.75	-	-	-	-
	Can you read and evaluate precautionary information in medication instructions?	938 (90.1)	0.72	-	-	-	Deleted^4^
	Can you adhere to prescription instructions for medication (frequency, dosage)?	985 (94.6)	0.76	-	-	-	Deleted^5^
Illnesses and treatment	Can you find information on the treatment or management of a concerning illness?	916 (88.0)	0.48	-	-	-	Deleted^4^
	Do you understand test and surgery results provided by hospitals?	916 (88.0)	0.59	-	-	-	Deleted^4^
	Can you manage post-treatment symptoms relief and side effect prevention on your own?	905 (86.9)	0.49	-	-	-	Deleted^3^
	Do you understand patient education materials provided by hospitals?^2^	897 (86.2)	0.62	-	-	-	-
Technology and resources							
Using health-related information	Do you understand health-related news on the internet or media (e.g., television, YouTube)?	912 (87.6)	0.40	0.63	-	-	Deleted^3^
	Do you understand how to find useful health information on the internet or media (e.g., television, YouTube)?	845 (81.2)	-	0.73	-	-	Deleted^5^
	Can you assess the reliability of health information obtained from the internet or media (e.g., television, YouTube)?^2^	707 (67.9)	-	0.57	-	-	-
	Can you apply health information obtained from the internet or media (e.g., television, YouTube) to actual health-related behaviors or decision making?^2^	815 (78.3)	-	0.67	-	-	-
	Can you find and utilize apps for health management?	765 (73.5)	-	0.76	-	-	Deleted^4^

1 Tthe percentage of both always and sometimes on 4 point Liker scale (1=never, 2=rarely, 3=sometimes, 4=always).

2 Values > 0.4 are presented, and final items. N(%) is the percentage of both always and sometimes on 4 point Liker scale (1=never, 2=rarely, 3=sometimes, 4=always).

3 Deleted based on factor loading.

4 Deleted based on item response theory.

5 Deleted based on expert review.

**Table 3.** Factor loading and reliability of the final HLIC (n=1,041)

Variables	Items	Mean±SD	Factor loading values^1^	Cronbach’s alpha coefficient
Disease prevention	Can you assess which vaccinations are necessary?	3.04±0.63	0.69	0.87
	Do you understand the degree of risk of mental health issues such as stress and depression?	2.95±0.67	0.70	
	Are you aware of the health symptoms resulting from excessive drinking, smoking, and lack of exercise?	2.99±0.62	0.65	
Health promotion	Can you evaluate how daily behaviors influence health?	2.99±0.61	0.67	
Healthcare	Do you have difficulty understanding explanations and instructions from doctors during medical appointments?	3.19±0.54	0.71	
	Can you assess what needs to be prioritized in case of an emergency?	2.93±0.69	0.68	
	Do you understand how to take medications as explained by doctors or pharmacists?	3.32±0.61	0.66	
	Do you understand patient education materials provided by hospitals?	3.09±0.64	0.72	
Technology and resources	Can you assess the reliability of health information obtained from the internet or media (e.g., television, YouTube)?	2.79±0.73	0.63	
	Can you apply health information obtained from the internet or media (e.g., television, YouTube) to actual health-related behaviors or decision making?	2.92±0.68	0.67	

HLIC, Health Literacy Index for the Community; SD, standard deviation.

1 4 point Likert scale (1=never, 2=rarely, 3=sometimes, 4=always).

**Table 4.** Correlation of the HLIC with the HLS-EU-Q16

HLS-EU-Q16		HLIC (10 items)									
		Disease prevention			Health promotion	Healthcare				Health-related resources	
		Vaccination	Mental health	Health risks	Healthy behavior	Communication with health professionals	Emergency	Medication	Illnesses and treatment	Information reliability	Media utilization
1.	Find information about treatments for illness that concern you?	0.39	0.40	0.41	0.40	0.38	0.39	0.38	0.42	0.39	0.45
2.	Find out where to get professional help when you are ill?	0.37	0.34	0.32	0.34	0.35	0.37	0.33	0.39	0.36	0.40
3.	Understand what your doctor tells you?	0.34	0.35	0.32	0.31	0.46^1^	0.38	0.40	0.43	0.33	0.37
4.	Understand your doctors’ or pharmacist’ instructions about how to take a prescribed medicine?	0.31	0.27	0.36	0.31	0.43	0.35	0.48^1^	0.41	0.25	0.37
5.	Judge when you many need to get a second opinion from another doctor?	0.31	0.37	0.28	0.32	0.33	0.40	0.25	0.31	0.41	0.37
6.	Use information the doctor gives you make decisions about your illness?	0.33	0.40	0.33	0.38	0.40^1^	0.39	0.39	0.44	0.37	0.45
7.	Follow instructions from your doctor or pharmacist?	0.32	0.32	0.35	0.33	0.44	0.32	0.42	0.40^1^	0.25	0.36
8.	Find information about how to manage mental health problems like stress or depression?	0.32	0.42^1^	0.35	0.38	0.32	0.40	0.29	0.38	0.42	0.41
9.	Understand health warnings about behavior such as smoking, low physical activity, and drinking too much?	0.34	0.41	0.47^1^	0.41	0.36	0.33	0.37	0.45	0.29	0.42
10.	Understand why you need health screenings?	0.37	0.37	0.39	0.37	0.45	0.33	0.43	0.41	0.35	0.40
11.	Judge if the information on health risks in the media is reliable?	0.31	0.36	0.27	0.32	0.27	0.38	0.25	0.32	0.54^1^	0.45
12.	Decide how you can protect yourself from illness based on information in the media?	0.35	0.36	0.33	0.36	0.30	0.34	0.28	0.33	0.47	0.44^1^
13.	Find out about activities that are good for your mental well-being?	0.31	0.36	0.38	0.35	0.36	0.35	0.38	0.40	0.29	0.43
14.	Understand advice on health from family members of friends?	0.34	0.32	0.35	0.36	0.42	0.32	0.38	0.40	0.35	0.43
15.	Understand information in the media about how to improve your health?	0.37	0.40	0.39	0.38	0.38	0.34	0.38	0.44	0.37	0.47
16.	Judge which everyday behavior is related to your health?	0.33	0.39	0.41	0.37^1^	0.34	0.33	0.35	0.39	0.39	0.44

HLIC, Health Literacy Index for the Community; HLS-EU-Q16, European Health Literacy Survey.

1 Items correspond to similar questions in the HLS-EU16.

**Table 5.** Comparison between the higher and lower scoring groups in the HLIC (n=1,041)

Variables	HLIC scores^1^		p-value
	<28	≥28	
Gender			0.523
Men (n=524)	112 (21.4)	412 (78.6)	
Women (n=517)	119 (23.0)	398 (77.0)	
Age (yr)			<0.001
19-29 (n=184)	34 (18.5)	150 (81.5)	
30-39 (n=185)	38 (20.5)	147 (79.5)	
40-49 (n=212)	34 (16.0)	178 (84.0)	
50-59 (n=210)	37 (17.6)	173 (82.4)	
≥60 (n=250)	88 (35.2)	162 (64.8)	
Education			<0.001
≤ Middle school (n=131)	71 (54.2)	60 (45.8)	
High school (n=323)	66 (20.4)	257 (79.6)	
College/University (n=498)	82 (16.5)	416 (83.5)	
Graduate school or higher (n=89)	12 (13.5)	77 (86.5)	
Monthly family income (US dollar)			<0.001
≤1,000 (n=50)	24 (48.0)	26 (52.0)	
1,000-<2,000 (n=109)	39 (35.8)	70 (64.2)	
2,000-<3,000 (n=157)	38 (24.2)	119 (75.8)	
3,000-<4,000 (n=136)	24 (17.6)	112 (82.4)	
4,000-<5,000 (n=126)	18 (14.3)	108 (85.7)	
≥5,000 (n=274)	43 (15.7)	231 (84.3)	

Values are presented as number (%). HLIC, Health Literacy Index for the Community.

1 Total score: 10 to 40, a high score denotes a high level of health literacy.

## Supplementary materials

Supplementary Material 1. Flow diagram for scoping review

Supplementary Material 2. Results of cognitive interview

Supplementary Material 3. ROC analysis for cutoff value

Supplementary Material 4. Final Korean version of the HLIC measurement instrument
