# Supplementary figures and images for: Development and validation of the Health Literacy Index for the Community for the Korean National Health and Nutrition and Examination Survey
Source: Epidemiol Health. 2024 Jul 10;46:e2024061. doi: 10.4178/epih.e2024061 (PMC11826031; doi:10.4178/epih.e2024061)

**Supplementary Material 1. Flow diagram for scoping review**


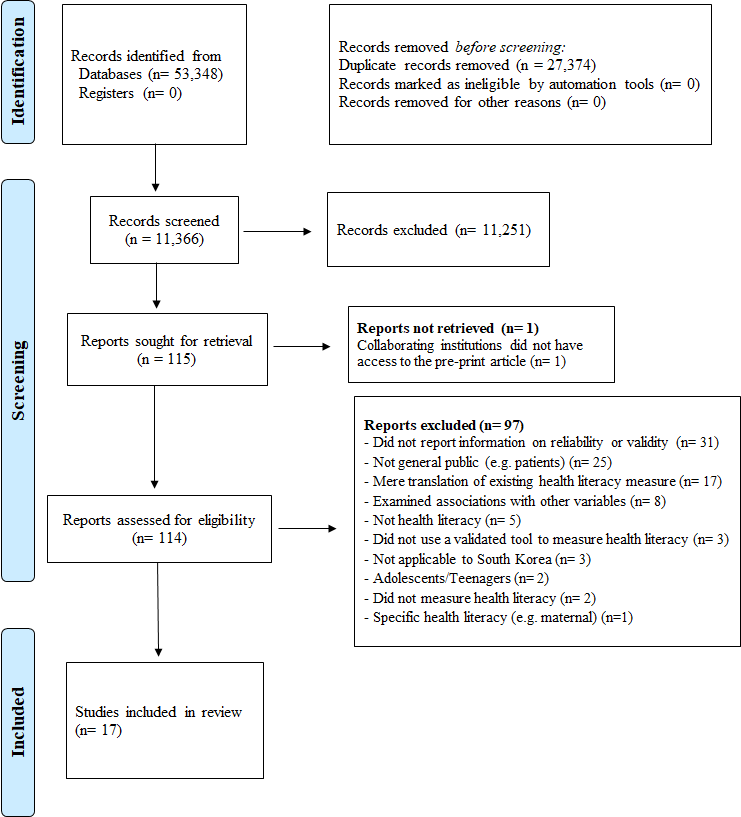

Supplement: Supplementary Material 1. — Flow diagram for scoping review [file epih-46-e2024061-Supplementary-1.docx]
