# Supplementary material for: Development and validation of the Health Literacy Index for the Community for the Korean National Health and Nutrition and Examination Survey
Source: Epidemiol Health. 2024 Jul 10;46:e2024061. doi: 10.4178/epih.e2024061 (PMC11826031; doi:10.4178/epih.e2024061)
Supplement: Supplementary Material 2. — Results of cognitive interview [file epih-46-e2024061-Supplementary-2.docx]

**Supplementary Material 2. Results of cognitive interview**

| **Item Number** | **Items (before revision)** | **Observations and comments** | **Changes** | **After cognitive interview**  **(revised version*)** |
| --- | --- | --- | --- | --- |
| **5** | Can you understand the degree of risk of mental health issues such as stress and depression? | Two people generally thought of mental health issues without personalizing them. | Inserted 'my' in front of ‘mental health’ | Can you understand the degree of risk of **your** mental health issues such as stress and depression? |
| **23** | Can you describe your current health status (e.g., medical history, medications)? | One person provided additional feedback. | Included 'symptoms' in the examples. | Can you describe your current health status (e.g., medical history, medications, **symptoms**)? |
| **27** | Can you find information on the treatment or management of health issues of interest? | Two people found the sentence somewhat difficult to understand. | Referred to similar HLS-EU items. | Can you find information on the treatment or management **of a concerning illness**? |

*Revised sections were highlighted in bold.
