# Supplementary material for: Development and validation of the Health Literacy Index for the Community for the Korean National Health and Nutrition and Examination Survey
Source: Epidemiol Health. 2024 Jul 10;46:e2024061. doi: 10.4178/epih.e2024061 (PMC11826031; doi:10.4178/epih.e2024061)
Supplement: Supplementary Material 4. — Final Korean version of the HLIC measurement instrument [file epih-46-e2024061-Supplementary-4.docx]

**Supplementary Material 4. Final Korean version of the HLIC measurement instrument**

- 다음은 평소 건강정보를 이해하고 활용할 때 겪을 수 있는 어려움에 대한 질문입니다. 귀하가 어려움을 느끼는 정도를 표시(√)해 주시기 바랍니다.

| 자가보고형 (10문항) | | **전혀 그렇지 않다** | **그렇지 않다** | **그렇다** | **매우 그렇다** |
| --- | --- | --- | --- | --- | --- |
| **1** | 필요한 예방 접종이 무엇인지 판단할 수 있습니까? | ① | ② | ③ | ④ |
| **2** | 나의 스트레스, 우울 증상과 같은 정신 건강 문제로 생기는 위험의 정도를 이해할 수 있습니까? | ① | ② | ③ | ④ |
| **3** | 과음, 흡연, 운동 부족으로 생길 수 있는 건강 이상 신호가 무엇인지 알고 있습니까? | ① | ② | ③ | ④ |
| **4** | 일상생활의 행동 가운데 어떤 것이 건강에 영향을 미치는지 판단할 수 있습니까? | ① | ② | ③ | ④ |
| **5** | 진료할 때 의사의 설명과 지시를 이해할 수 있습니까? | ① | ② | ③ | ④ |
| **6** | 응급 상황이 생겼을 때 먼저 해야 할 일이 무엇인지 판단할 수 있습니까? | ① | ② | ③ | ④ |
| **7** | 의사나 약사가 설명해 주는 약 먹는 방법을 이해할 수 있습니까? | ① | ② | ③ | ④ |
| **8** | 병원에서 받은 환자용 교육 자료를 이해할 수 있습니까? | ① | ② | ③ | ④ |
| **9** | 인터넷이나 미디어에서 얻은 건강 정보가 믿을 만한 것인지 판단할 수 있습니까? | ① | ② | ③ | ④ |
| **10** | 인터넷이나 미디어에서 얻은 건강 정보를 건강과 관련한 행동이나 의사결정에 활용할 수 있습니까? | ① | ② | ③ | ④ |
